# Diagnostic value of ultrasound radiomic features in differentiating benign and malignant breast lesions

**DOI:** 10.1007/s40477-025-01025-8

**Published:** 2025-06-27

**Authors:** Yuke Gong, Yan Cheng, Yan Liu, Guohui Zhang, Shuang Li, Ruiqi Wu, Hongmei Wang, Lizhou Lu

**Affiliations:** 1 Ultrasound Medicine Major (Graduate School), Qujing Affiliated Hospital of Kunming Medical University, Qujing, Yunnan China; 2 Department of Ultrasound, Dali Prefecture People’s Hospital, Dali, Yunnan China; 3 Department of Ultrasound, Qujing Affiliated Hospital of Kunming Medical University, Qujing, Yunnan China; 4 Department of Ultrasound, Qilin District People’s Hospital, Qujing, Yunnan China; 5 Department of Ultrasound, The First People’s Hospital of Zhaotong, Zhaotong, Yunnan China; 6 Department of Ultrasound, The First Hospital of Yunnan Province, Kunming, Yunnan China

**Keywords:** Ultrasound, Breast cancer, Radiomics, Semantic features

## Abstract

**Purpose:**

This study aims to explore the relationship between ultrasound radiomics features and semantic features from BI-RADS classification in the preoperative differentiation of benign and malignant breast lesions, as well as the potential diagnostic advantages of radiomics features.

**Methods:**

Retrospective analysis was performed on 147 female patients with pathologically confirmed breast lesions. Ultrasound images and clinical data were used to construct three diagnostic models: BI-RADS classification single factor diagnostic model, Radiomics diagnostic model, and a BI-RADS-radiomic combined model. Additionally, univariate radiomic models based on semantic features were developed to investigate the associations.

**Results:**

The BI-RADS-Radiomics combined model demonstrated superior performance in both training and testing sets, with AUC values of 0.985 and 0.964, respectively. It also exhibited optimal diagnostic consistency and clinical net benefit. Significant correlations were observed between multiple radiomics features and specific semantic features (AUC range: 0.609–0.752).

**Conclusion:**

Radiomics features effectively assist in breast cancer diagnosis via ultrasound and exhibit nonlinear associations with specific semantic features.

**Supplementary Information:**

The online version contains supplementary material available at 10.1007/s40477-025-01025-8.

## Introduction

As one of the core tools of breast screening, ultrasound standardizes the diagnosis of breast masses according to BI-RADS classification [1], but due to the subjectivity of doctors'operation [2], the misdiagnosis rate of breast masses containing cross ultrasound signs [3] can reach 30%, and the accurate diagnosis of some masses is still a challenge [4]. Radiomics extract features from medical images by quantitative means for analysis, which provides a promising solution to improve objectivity [5], but they lack interpretability. It is difficult in practical clinical decision-making applications, and doctors are difficult to make decisions directly based on radiomics features. Therefore, this study attempts to combine the two to build a joint model, and establish an univariate imaging radiomics model based on ultrasound semantic features, aiming to improve the diagnostic performance and the interpretability of radiomics features.

## Materials and methods

### Ultrasonography and semantic feature assessment

Based on the above research background, this study constructs a diagnostic model to verify the hypothesis by retrospectively analyzing patient data. A total of 147 female patients who underwent breast ultrasound examination in the ultrasound department of Qujing Affiliated Hospital of Kumming Medical University from June 2022 to July 2023 were collected, and 328 ultrasound images were obtained. All data were approved by the hospital ethics committee, exempted from informed consent, and images were anonymized to ensure patient privacy [6]. Inclusion criteria: clear pathological results; Female patients aged 18–80 years; BI-RADS class 2–5. Exclusion criteria: images with poor quality and more interference information.

In this study, GE—LOGIQ E20 was used for examination. Patients were placed in supine position, and the maximum transverse section of lesion diameter was obtained for image storage. BI-RADS classification and the following six features were independently evaluated by two sonographer with more than 5 years of working experience according to the guidelines and specifications for breast cancer diagnosis and treatment of the China anticancer association (2024 version) [7]: shape (regularity/irregularity), aspect ratio (transverse/longitudinal), boundary (clear/unclear), edge (smooth/irregular), internal echo (even/uneven), calcification (yes/no). In case of disagreement, a third senior sonographer will comprehensively analyze the evaluation opinions, original ultrasound images and relevant clinical data of the first two sonographers. If the result is consistent with one of the first two, the result will prevail; if the evaluation result of the third sonographer is different from the former two, the evaluation result shall be finally determined through internal discussion of the Department. To facilitate the evaluation of the performance of the BI-RADS group, the benign and malignant cut-off value was set as BI-RADS 4a [8].

### Radiomic feature extraction and model construction

#### Lesion delineation and pretreatment

All lesion pictures were imported into Darwin scientific research platform (http://premium.darwin.yizhun-ai.com), normalized to eliminate differences in instrument parameters. The region of interest (ROI) is independently outlined by a sonographer with more than five years of work experience, and then confirmed by a senior sonographer.

#### Feature extraction

With the benign and malignant pathological results as the label, all radiomics features are exported to R software from Darwin scientific research platform. In the export process, it is necessary to ensure the integrity of the data and avoid data loss or error. Stratified random sampling was carried out according to the ratio of 5:2 (to ensure the consistent proportion of benign and malignant), which was divided into training set (103 cases) and testing set (44 cases). Significant features were screened by univariate analysis (*t* test/Mann–Whitney *U* test, *p* < 0.05), redundant features were further removed by mutual information (mutual information value > 0) and Spearman correlation analysis (correlation coefficient < 0.9), and finally the dimension was reduced by lasso regression (*λ* value was selected by 10-fold cross validation), and the key features were finally screened.

### Model construction and performance evaluation

Taking the benign and malignant pathological results as the label, the model was constructed using the two-way stepwise regression method [9]. The entry criteria of variables were *p* < 0.05, and the exclusion criteria were *p* > 0.05 (Appendix Table S3):

Model 1: BI-RADS classification single factor diagnostic model;

Model 2: Radiomics diagnostic model;

Model 3: BI-RADS-Radiomics combined diagnostic model.

Taking pathological results as the gold standard, the area under the receiver operating characteristic (AUC), sensitivity, specificity, calibration curve and decision curve analysis (DCA) were used to evaluate the performance.

### Analysis of the relationship between radiomics features and semantic features

Semantic features were used as the classification criteria, and"irregular shape, longitudinal, unclear, uneven, with calcification"was set as the positive category (Appendix Table S2). Univariate Radiomics models were constructed one by one with radiomics features as the dependent variable. By calculating AUC of the model, the relationship between radiomics features and corresponding semantic features was evaluated. At the same time, boxplots of some significant radiomics features in the two categories of semantic features are drawn, and also their descriptive statistics such as the mean and standard deviation in different semantic feature categories are calculated, so as to show the differences more intuitively.

### Statistical methods

R software version 4.3.2 and python software version 3.9 were used. Quantitative variables were reported as mean ± standard deviation or median (25 th percentile, 75 th percentile), and normality and homoscedasticity were tested using Kolmogorov–Smirnov and Bartlett test, respectively. Independent samples *t*-test or Mann–Whitney *U* test was used to analyze quantitative variables, and Chi square test or Fisher exact test was used to analyze categorical data. Statistical significance was set at two-sided *p* < 0.05.

## Result

Finally, 147 patients and 328 pictures were included, including 93 patients with benign pathological results and 54 patients with malignant pathological results. There were significant differences in key ultrasound features (shape, aspect ratio, boundary, edge, calcification) between benign and malignant groups (*p* > 0.05) (Appendix, Table S1).

**Fig. 1:**
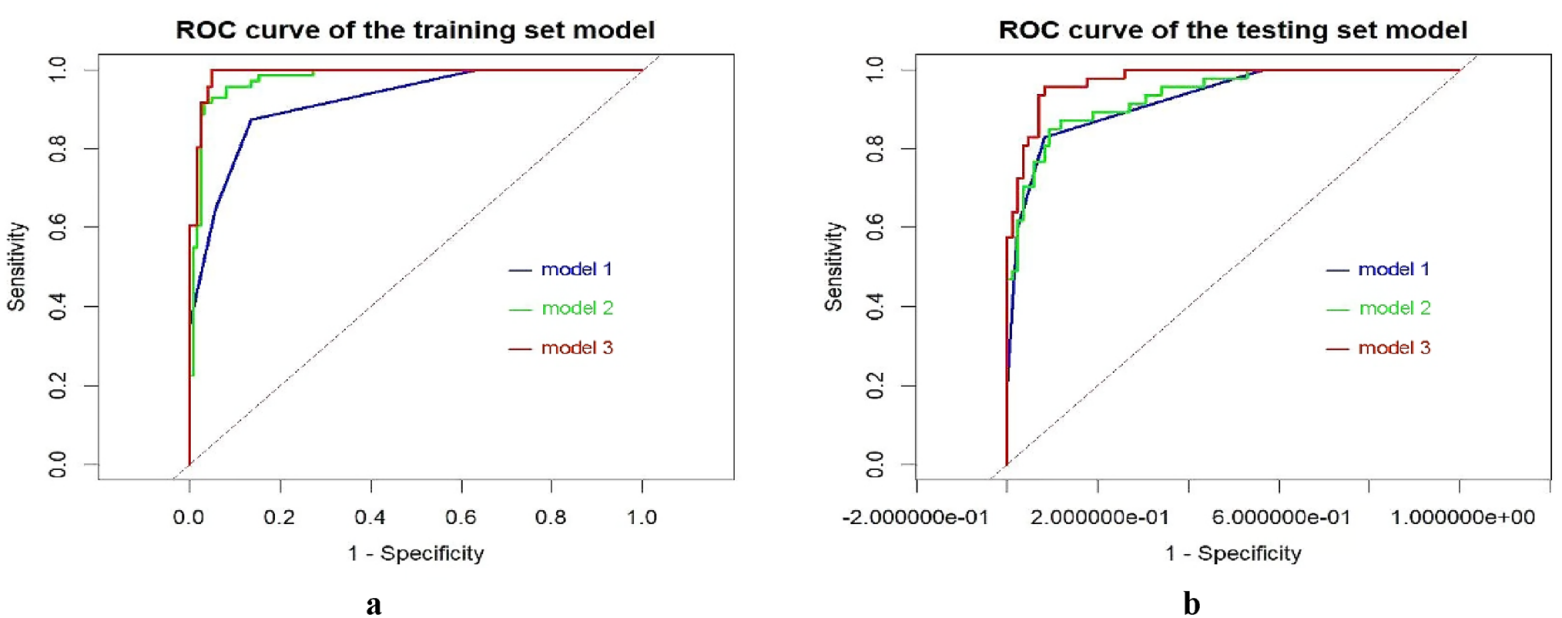
*Receiver operating characteristic (ROC)* curves of prediction model in training set (**a**) and testing set (**b**)

**Fig. 2:**
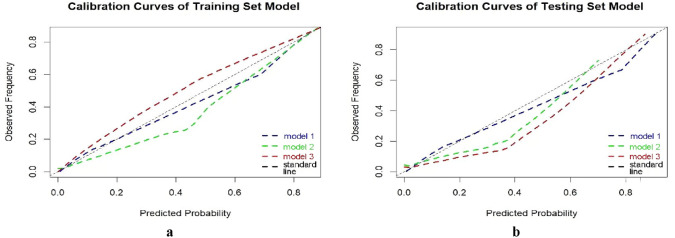
*Calibration curve* between model predicted probability and actual probability in training set (**a**) and testing set (**b**). The *x-axis* is the predicted probability of the model, and the *y-axis* is the actual occurrence; The dashed line represents the ideal reference line, and the blue solid line is the prediction curve of the prediction model

**Fig. 3:**
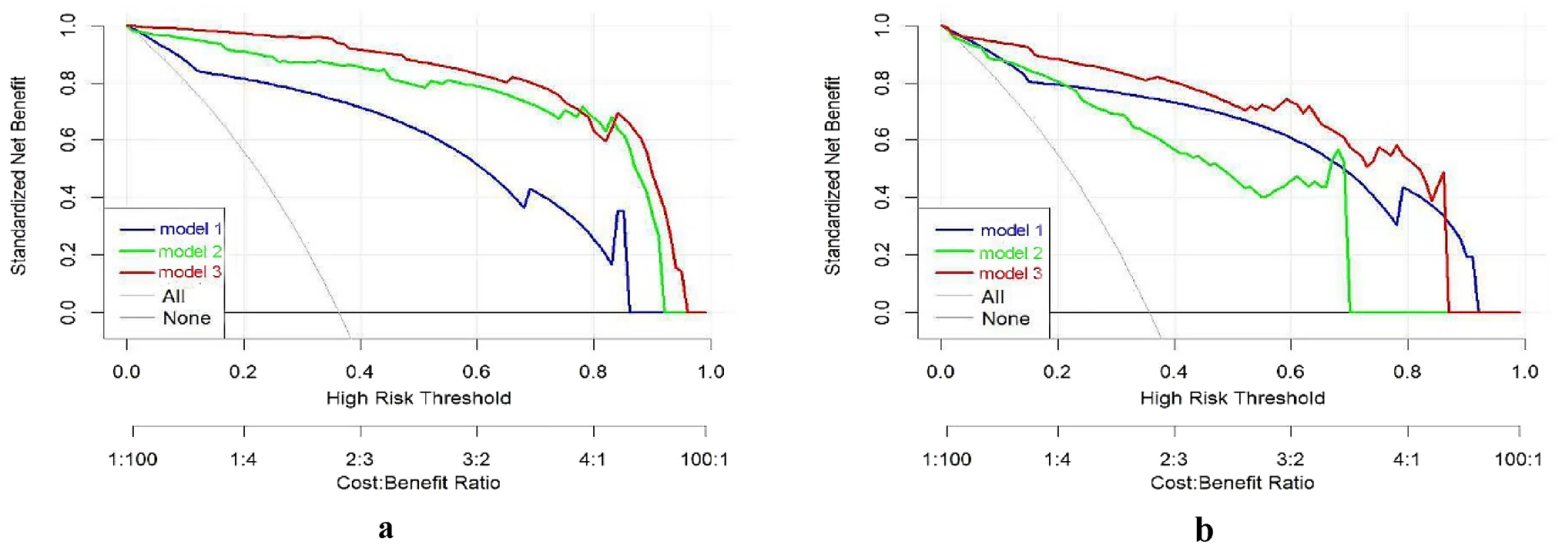
Analysis of model *decision curve (DCA)* in training set (**a**) and testing set (**b**). *Y-axis* measures net benefit. The net benefit was determined by calculating the difference between the expected benefit and the expected harm associated with each proposed model [net benefit = true positive rate − (false positive rate × weighting factor), weighting factor = threshold probability/(1 − threshold probability)]. The gray curve represents the assumption that all lesions are malignant, and the black horizontal line represents the assumption that all lesions are benign. When the threshold probability is > 5%, the benefit of using the joint model to predict malignancy is greater than the other two models

In the training set and testing set, the comprehensive performance (Fig. 1) and clinical net benefit of model 3 were the best (Fig. 2), AUC: 0.985 (training) and 0.964 (testing), and the sensitivity (0.865) and specificity (0.975) in the testing set were better than the single model (Table 1). The calibration curve showed that the prediction of malignant tumors by the three models was in good agreement with the pathological results (Fig. 3).

**Table 1:** Diagnostic performance of multivariate logistic regression model of each model

	Index	Model 1	Model 2	Model 3
Training set	AUC (95%CI)	0.904 [0.886–0.921]	0.969 [0.956–0.978]	0.985 [0.981–0.990]
	ACC	0.867	0.949	0.969
	SE	0.785	0.942	0.922
	SP	0.923	0.953	1
	BT	0.305	0.98	0.884
	PPV	0.873	0.915	1
	NPV	0.864	0.968	0.952
Testing set	AUC (95%CI)	0.907 [0.887–0.928]	0.915 [0.894–0.936]	0.964 [0.954–0.975]
	ACC	0.886	0.886	0.932
	SE	0.848	0.833	0.865
	SP	0.907	0.917	0.975
	BT	0.393	0.986	0.892
	PPV	0.83	0.851	0.957
	NPV	0.918	0.906	0.918

*ACC* accuracy, *SEN* sensitivity, *SPE* specificity, *BT* best threshold, *PPV* positive predictive value, *NPV* negative predictive value, *95%CI* indicates 95% confidence interval

**Fig. 4:**
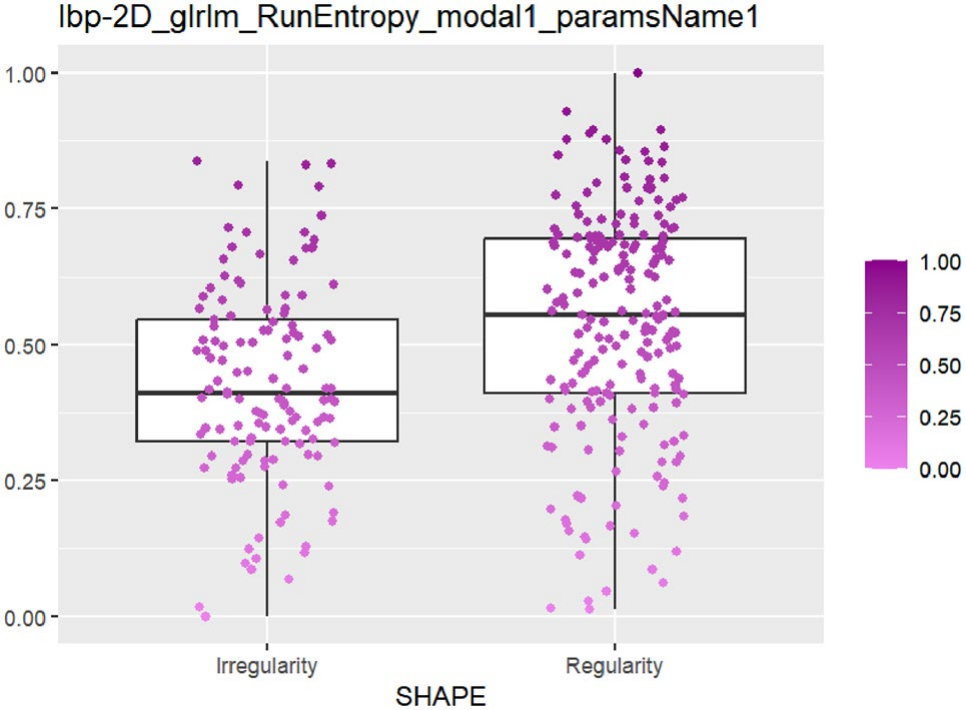
The boxplot of lbp-2D_glrlm_RunEntropy_modal1 and shape features (left: irregular shape group, right: regular shape group)

**Fig. 5:**
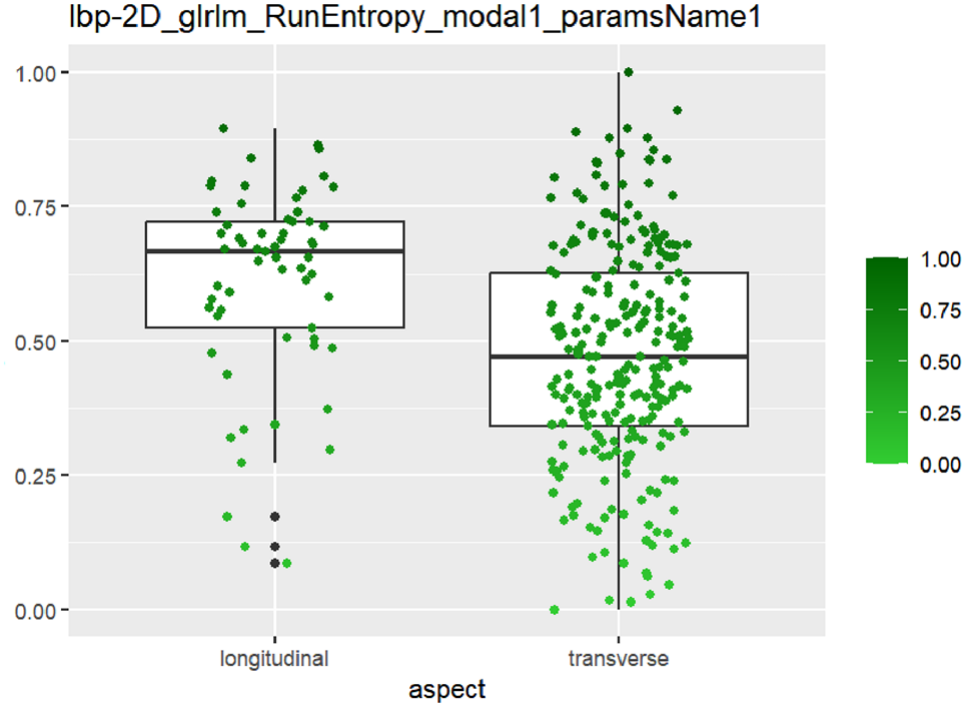
The boxplot of lbp-2D_glrlm_RunEntropy_modal1 and aspect ratio features (left: longitudinal group, right: transverse group)

**Fig. 6:**
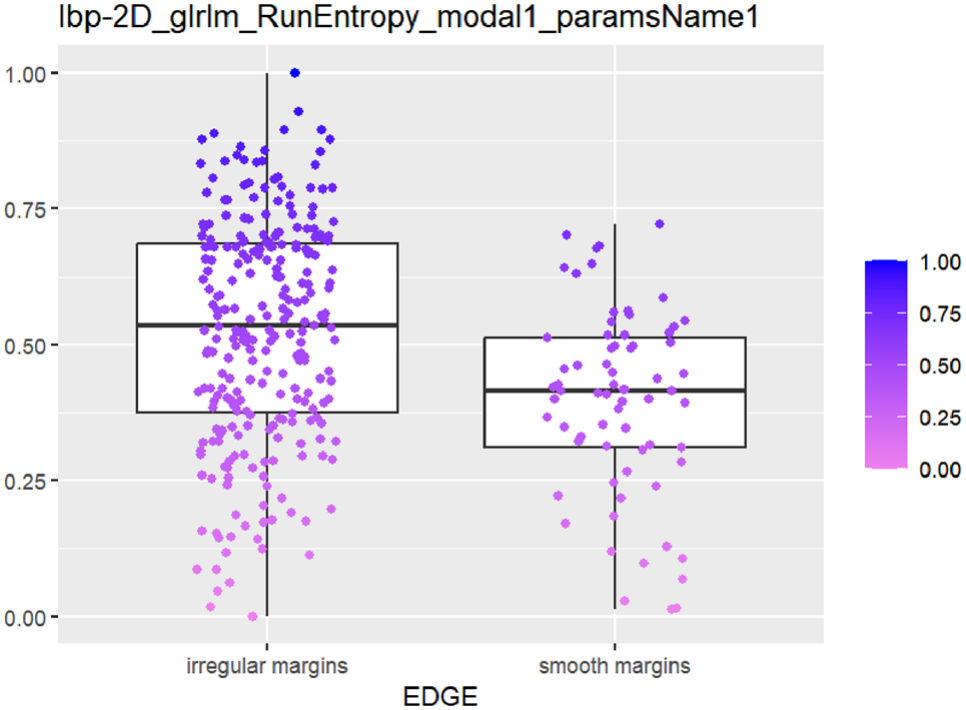
The boxplot of *lbp-2D_glrlm_RunEntropy_modal1* and edge features (left: edge irregular group, right: edge smoothing group)

**Fig. 7:**
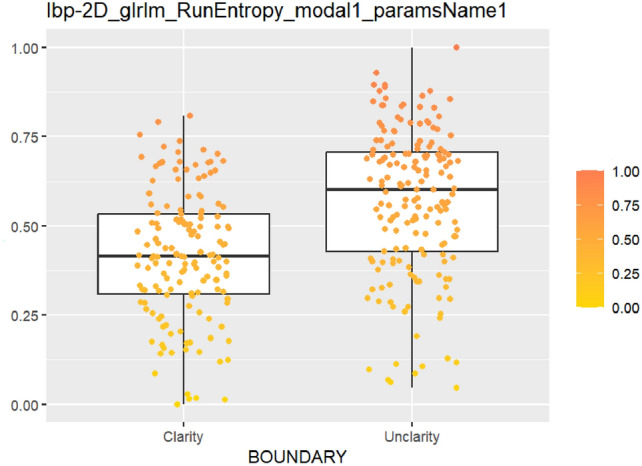
The boxplot of *lbp-2D_glrlm_RunEntropy_modal1* and boundary features (left: clear boundary group, right: unclear boundary group)

**Fig. 8:**
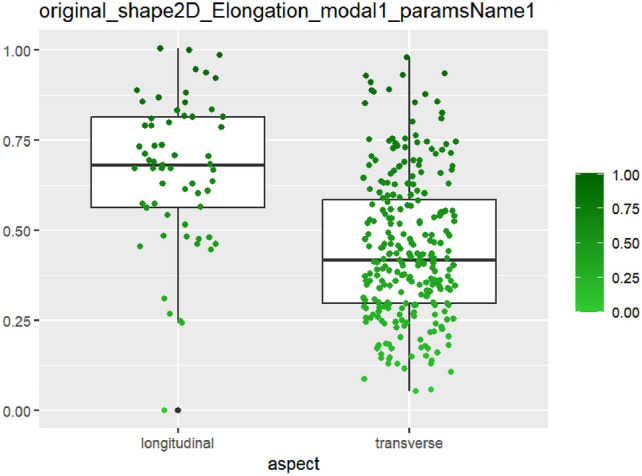
The boxplot of *original_shape2D_Elongation_moda-l1* and aspect ratio characteristics (left: longitudinal group, right: transverse group)

**Fig. 9:**
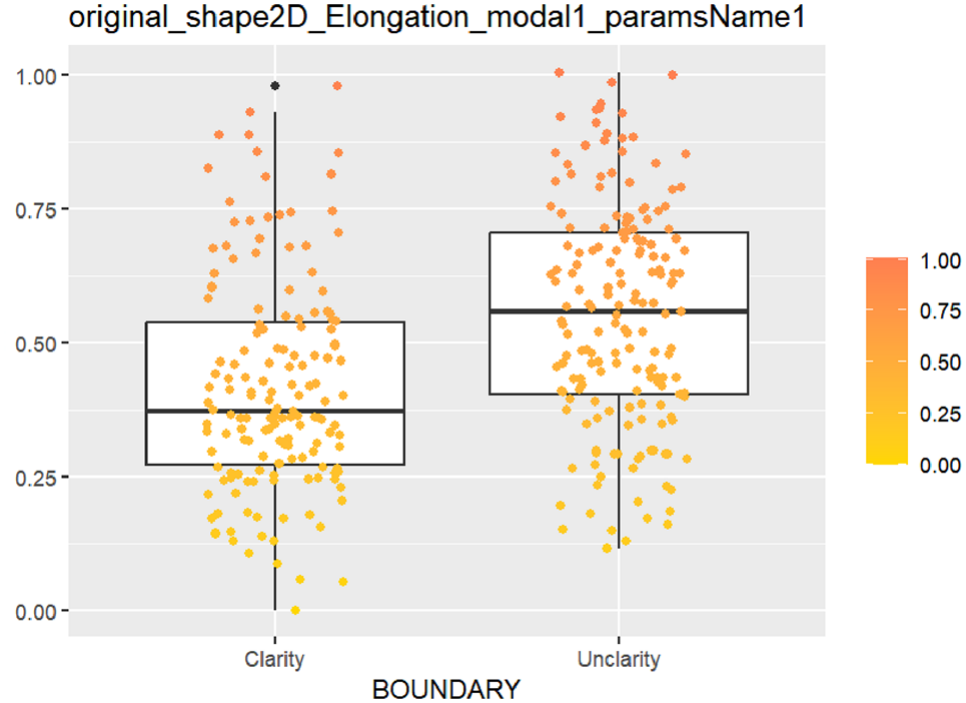
The boxplot of *original_shape2D_Elongation_mo-dal1* and boundary features (left: clear boundary group, right: unclear boundary group)

After feature selection and dimension reduction, 13 features were obtained. After single factor analysis, it was found that there was a certain relationship with specific semantic features. The texture feature RunEntropy (RE) is correlated with irregular shape, longitudinal growth, unclear boundary and uneven edge (AUC = 0.637–0.692) (Figs. 4, 5, 6, 7, Appendix Tables S7–10). Shape feature Elongation is correlated with aspect ratio and unclear boundary (AUC = 0.752, 0.656) (Figs. 8, 9; Appendix Tables S8, 9). Shape and Busyness (AUC = 0.651); Aspect ratio and GrayLevelVariance (AUC = 0.609); Boundary and 10Percentile (AUC = 0.616). AUC of all radiomics features showed general performance in calcification (AUC range: 0.454–0.571), but the sensitivity of busyness, rMAD and 10Percentile was higher (0.723–0.877), and the specificity of RE and DV was higher (0.788, 0.732), suggesting that the heterogeneity of calcification needs to be combined with multi feature analysis (Table 1).

## Discussion

The above results show that the joint model is significantly better than the single model in the diagnostic performance. To further explore its clinical significance, this study combines the relationship between semantic features and radiomics features to carry out the following discussion, which shows that the establishment of single factor model provides specific data support and new ideas for the interpretability of radiomics features. Radiomics makes up for the subjective limitations of semantic features by quantifying tumor features. RE reflects the randomness of gray distribution, which is consistent with the disordered growth pattern of malignant tumors. In previous studies [10], it can be used as an important predictor of recurrence free survival rate of patients with primary breast cancer. In the task of distinguishing benign lesions from Luminal-A breast cancer [11] by using linear discriminant analysis classifier, RE obtained 72.9% of AUC, which shows it as an independent predictor, the potential of classification of benign and malignant tumors needs to be tapped. Elongation (range 0–1), converging to 1 indicates that ROI is close to a circle, and to 0 indicates that it is close to a straight line. The high weight of it can objectively explain that malignant tumors with longitudinal growth tendency squeeze the surrounding tissues to different degrees, which is consistent with the biological behavior of malignant lesions that break through the tissue plane [12] and show linear migration and different cell growth rates reported in the literature [13].

In this study, apart from RE and Elongation, the other features are related to only one kind of semantic features: Busyness and irregular shape, GrayLevelVariance (GLV) and longitudinal, 10Percentile and unclear boundary. Busyness refers to the measure of pixel change. GLV measures the variance of gray value. Both are texture features, with high values and strong heterogeneity. In the early stage, we subjectively believed that the results of the two will perform well in categories of echo pattern group, but there was no statistical difference in the single factor analysis of the index, which means the group can not be included in the subsequent analysis. Huang [14] has pointed out that ER, PR(+) and TNBC are mostly hyperechoic or mixed echo, while Luminal-B type is mostly hypoechoic, and the diagnosis cannot rely solely on the internal echo pattern. Although Busyness and GLV have no direct connection with the expected sematic feature, they still provide a side reference, that is, the echo pattern of the lesion with unclear boundary and longitudinal growth is more heterogeneous, which is the same as Song's view [15]: a large number of cells in the malignant tumor are closely arranged, and the growth rate is different, causing the imbalance of peripheral growth and easy to form concave convex grooves. 10Percentile refers to the gray value of 10%, which is positively correlated with unclear boundary or the lack of interstitial structure with cancer cells, and it is very easy to diffuse to the surrounding tissues, forming part of the blurred boundary on the sonogram. In ultrasound, it is mostly low or no echo, with blurred boundary and interrupted echo.

Features like RE, Elongation provided a more detailed analysis of tissue properties using quantitative image information [16], confirming that irregular shape, longitudinal growth, unclear boundary, and uneven edge can be the core ultrasound signs of malignant lesions, which is consistent with the previous research’s point [17], that breast masses containing such signs should be set as high-risk group, besides the follow-up time should be extended, and clinical treatment should be carried out in advance [18]. In the calcification category, the overall radiomics features are generally expressed. Although some features have high sensitivity or specificity, the sample size of calcification group still needs to be expanded to explain this phenomenon because the sample size of the two categories is similar.

The radiomics model (model 2) composed of 13 robust features can successfully classify benign and malignant breast lesions, and the model (model 3) composed of 9 robust features combined with BI-RADS classification significantly improves the diagnostic accuracy of breast lesions, which indicates that radiomics features as an effective complement of semantic features have good value in the prediction of breast cancer, and the combination of the two can effectively improve the diagnostic accuracy and clinical applicability. This is similar to the conclusions of previous studies [19, 20]. Radiomics serves as an auxiliary tool for traditional imaging by capturing key malignant tumor features (e.g. texture heterogeneity and morphology). For instance, texture features such as RE [21] quantify tumor heterogeneity through extensive parameter analysis, providing radiologists with imperceptible intratumoral information. Morphological features like Elongation are directly related to aspect ratio and boundary. Several experiments [22, 23] in the field of tumor imaging have shown that it may be beneficial to combine morphological features extracted from ROI with established diagnostic practice, which can standardize the evaluation of lesions, and improve the consistency of collaborative diagnosis based on BI-RADS [24]. Perhaps in the future, AI diagnosis model based on radiomics will be incorporated into the clinic, and shape features will be the most intuitive and easily accepted.

Whether radiomics features can be used alone as an independent predictor of malignancy, and the integrated model can refine the diagnostic path for different patient populations while improving the diagnostic accuracy, so as to specify personalized diagnosis and treatment plans, for example, there are differences in the biological behavior and treatment response of different molecular types of breast cancer [25]. Radiomics may have unique significance in evaluating tumor aggressiveness and prognosis. There are also some limitations in this study, such as the relatively small sample size and the inclusion of only a single ultrasonic mode, which may affect the universality of the conclusion. In future research, our case collection can be expanded to include patients with different regions and ultrasound manifestations; integrate multimodal imaging (such as elastography, angiography), complete medical history, molecular markers and other clinical information; The external testing set should be added to enhance the model generalization ability, further improve the diagnostic performance and apply to multiple scenarios.

## Conclusion

Radiomics features not only have independent diagnostic potential, but also can significantly improve the diagnostic ability of ultrasound breast benign and malignant lesions model based on semantic features. The integration of the two can synergistically optimize the clinical prediction model and jointly support accurate classification and decision-making.

## Supplementary Information

Below is the link to the electronic supplementary material.

Supplementary file1 (DOCX 51 KB)

## Data Availability

All clinical data in this study were obtained from self funded ultrasound examinations by patients, and no sponsorship project funding was applied for or used.
