# Supplementary material for: Diagnostic value of ultrasound radiomic features in differentiating benign and malignant breast lesions
Source: J Ultrasound. 2025 Jun 27;28(3):645–52. doi: 10.1007/s40477-025-01025-8 (PMC12496310; doi:10.1007/s40477-025-01025-8)
Supplement: Supplementary file 1 — Supplementary file1 (DOCX 51 KB) [file 40477_2025_1025_MOESM1_ESM.docx]

| **Table S1.**  Single factor analysis of semantic features and BI-RADS classification | | | | | | |
| --- | --- | --- | --- | --- | --- | --- |
| **Characteristics** | **Training set**  **(n=197)** | | ***p*-value** | **Testing set**  **(n=131)** | | ***p-value*** |
|  | **Benign**  **(n=126)** | **Malignant (n=71)** |  | **Benign (n=84)** | **Malignant**  **(n=47)** |  |
| ***Shape* *（%）*** |  |  |  |  |  |  |
| Regularity | 53（42.1） | 69（97.2） | ＜0.05 | 37（44.0） | 43（91.5） | ＜0.05 |
| Irregularity | 73（57.9） | 2（2.8） |  | 47（56.0） | 4（8.5） |  |
| ***Boundary*  *（%）*** |  |  |  |  |  |  |
| Clear | 80（63.5） | 6（8.5） | ＜0.05 | 59（70.2） | 10（21.3） | ＜0.05 |
| Unclear | 46（36.5） | 65（91.5） |  | 25（29.8） | 37（78.7） |  |
| ***Edge（%）*** |  |  |  |  |  |  |
| Smooth | 43（34.1） | 0 | ＜0.05 | 24（28.6） | 2（4.3） | ＜0.05 |
| Irregular | 83（65.9） | 71（100%） |  | 60（71.4） | 45（95.7） |  |
| ***Echo pattern （%）*** |  |  |  |  |  |  |
| Even | 102（80.9） | 52（73.2） | ＞0.05 | 64（76.2） | 37（78.7） | ＞0.05 |
| Uneven | 24（19.1） | 19（26.8） |  | 20（23.8） | 10（21.3） |  |
| ***Aspect ratio （%）*** |  |  |  |  |  |  |
| transverse （<1） | 118（93.7） | 42（59.2） | ＜0.05 | 78（92.9） | 29（61.7） | ＜0.05 |
| longitudinal （>1） | 8（6.3） | 29（40.8） |  | 6（7.1） | 18（38.3） |  |
| ***Calcification* （%）** |  |  |  |  |  |  |
| Absent | 91（72.2） | 43（60.6） | ＜0.05 | 58（69.0） | 21（44.7） | ＜0.05 |
| Present | 35（27.8） | 28（39.4） |  | 26（31.0） | 26（55.3） |  |
| ***BI-RADS***  ***Final assessment （%）*** |  |  |  |  |  |  |
| 2 | 1（0.7） | 0 | ＜0.05 | 0 | 0 | ＜0.05 |
| 3 | 47（37.3） | 0 |  | 36（42.9） | 0 |  |
| 4a | 62（49.3） | 9（12.7） |  | 41（48.8） | 8（17.0） |  |
| 4b | 10（7.9） | 16（22.5） |  | 5（6.0） | 11（23.4） |  |
| 4c | 6（4.8） | 21（29.6） |  | 2（2.4） | 19（40.4） |  |
| 5 | 0 | 25（35.2） |  | 0 | 9（19.1） |  |

| **Table S2.**  Single factor analysis of semantic features and radiomics features | | |
| --- | --- | --- |
| **Feature** | ***p*-value** |  |
| BI-RADS | ＜0.001 |  |
| Shape | ＜0.001 |  |
| Aspect ratio | ＜0.001 |  |
| Boundary | ＜0.001 |  |
| Edge | ＜0.001 |  |
| Calcification | ＜0.001 |  |
| Echo pattern | ＞0.05 |  |
| original_shape2D_Elongation_modal1 | ＜0.001 |  |
| original_shape2D_Sphericity_modal1 | ＜0.001 |  |
| gradient_glrlm_RunVariance_modal1 | 0.029 |  |
| gradient_ngtdm_Busyness_modal1 | ＜0.001 |  |
| lbp-2D_firstorder_RobustMeanAbsoluteDeviation_modal1 | ＜0.001 |  |
| lbp-2D_glrlm_RunEntropy_modal1 | ＜0.001 |  |
| squareroot_glrlm_GrayLevelNonUniformityNormalized_modal1 | 0.007 |  |
| wavelet-LH_gldm_DependenceVariance_modal1 | 0.002 |  |
| wavelet-LH_glszm_LargeAreaHighGrayLevelEmphasis_modal1 | ＜0.001 |  |
| wavelet-HL_gldm_SmallDependenceEmphasis_modal1 | ＜0.001 |  |
| wavelet-HL_glszm_GrayLevelVariance_modal1 | ＜0.001 |  |
| wavelet-LL_firstorder_10Percentile_modal1 | 0.012 |  |
| log-sigma-3-0-mm-3D_firstorder_Maximum_modal1 | 0.002 |  |

| **Table S3.**  Formula of each model | |
| --- | --- |
| **Model** | **Formula** |
| Model 1: BI-RADS classification single factor diagnostic model | -1.805+ 1.893×BI-RADS |
| Model 2: Radiomics diagnostic model | Radscore = -4.129+6.408×original_shape2D_Elongation_modal1-8.954×original_shape2D_Sphericity_modal1-3.994×gradient_glrlm_RunVariance_modal1+3.058×gradient_ngtdm_Busyness_modal1+6.27×lbp-2D_firstorder_RobustMeanAbsoluteDeviation_modal1+8.456×lbp-2D_glrlm_RunEntropy_modal1-3.523×squareroot_glrlm_GrayLevelNonUniformityNormalized_modal1-3.627×wavelet-LH_gldm_DependenceVariance_modal1_-1.904×wavelet-LH_glszm_LargeAreaHighGrayLevelEmphasis_modal1_-5.105×wavelet-HL_gldm_SmallDependenceEmphasis_modal1_-4.988×wavelet-HL_glszm_GrayLevelVariance_modal1_+3.689×wavelet-LL_firstorder_10Percentile_modal1+7.996×log-sigma-3-0-mm-3D_firstorder_Maximum_modal1 |
| Model 3: BI-RADS-Radiomics combined diagnostic model | Radscore = -3.002 +1.678*BI-RADS+6.176×original_shape2D_Elongation_modal1-8.213×original_shape2D_Sphericity_modal1-6.573×gradient_glrlm_RunVariance_modal1+4.37×lbp-2D_firstorder_RobustMeanAbsoluteDeviation_modal1+10.221×lbp-2D_glrlm_RunEntropy_modal1-4.411×squareroot_glrlm_GrayLevelNonUniformityNormalized_modal1-6.456×wavelet-HL_gldm_SmallDependenceEmphasis_modal1-3.721×wavelet-HL_glszm_GrayLevelVariance_modal1+2.384×wavelet-LL_firstorder_10Percentile_modal1 |

| **Table S4.** Logistic regression parameters of BI-RADS classification single factor diagnostic model (model 1) | | | | | |
| --- | --- | --- | --- | --- | --- |
| **Feature** | **Estimate** | **Std. Error** | **z-value** | **Pr(>\|z\|)** |  |
| Intercept | -1.805 | 0.225 | -8.033 | ＜0.001 |  |
| BI_RADS | 1.893 | 0.204 | 9.266 | ＜0.001 |  |
| *Intercept* is the cut-off value of the correction model and does not participate in VIF calculation; *Estimate* is the coefficient estimate of each independent variable, indicating the influence degree of the independent variable on the dependent variable | | | | |  |

| **Table S5.** Logistic regression parameters of Radiomics diagnostic model (model 2) | | | | | | |
| --- | --- | --- | --- | --- | --- | --- |
| **Feature** | **Estimate** | **Std. Error** | **z-value** | **Pr(>\|z\|)** | **VIF** |  |
| Intercept | -4.129 |  |  |  |  |  |
| original_shape2D_Elongation_modal1 | 6.408 | 1.887 | 3.396 | 0.001 | 3.086 |  |
| original_shape2D_Sphericity_modal1 | -8.954 | 2.297 | -3.898 | 0.000 | 2.726 |  |
| gradient_glrlm_RunVariance_modal1 | -3.994 | 2.307 | -1.731 | 0.083 | 1.641 |  |
| gradient_ngtdm_Busyness_modal1 | 3.058 | 2.177 | 1.405 | 0.160 | 3.401 |  |
| lbp-2D_firstorder_RobustMeanAbsoluteDeviation_modal1 | 6.270 | 1.778 | 3.527 | 0.000 | 1.386 |  |
| lbp-2D_glrlm_RunEntropy_modal1 | 8.456 | 3.104 | 2.725 | 0.006 | 3.940 |  |
| squareroot_glrlm_GrayLevelNonUniformityNormalized_modal1 | -3.523 | 1.756 | -2.007 | 0.045 | 1.371 |  |
| wavelet-LH_gldm_DependenceVariance_modal1 | -3.627 | 1.578 | -2.298 | 0.022 | 3.203 |  |
| wavelet-LH_glszm_LargeAreaHighGrayLevelEmphasis_modal1 | -1.904 | 1.145 | -1.663 | 0.096 | 1.349 |  |
| wavelet-HL_gldm_SmallDependenceEmphasis_modal1 | -5.105 | 3.033 | -1.683 | 0.092 | 1.379 |  |
| wavelet-HL_glszm_GrayLevelVariance_modal1 | -4.988 | 1.866 | -2.673 | 0.008 | 1.224 |  |
| wavelet-LL_firstorder_10Percentile_modal1 | 3.689 | 1.011 | 3.650 | 0.000 | 1.376 |  |
| log-sigma-3-0-mm-3D_firstorder_Maximum_modal1 | 7.996 | 2.846 | 2.810 | 0.005 | 3.369 |  |
| *Intercept* is the cut-off value of the correction model and does not participate in VIF calculation; *Estimate* is the coefficient estimate of each independent variable, indicating the influence degree of the independent variable on the dependent variable | | | | | |  |

| **Table S6.**  Logistic regression parameters of BI-RADS-Radiomics combined diagnostic model (model 3) | | | | | | |
| --- | --- | --- | --- | --- | --- | --- |
| **Feature** | **Estimate** | **Std. Error** | **z-value** | **Pr(>\|z\|)** | **VIF** |  |
| Intercept | -3.002 |  |  |  |  |  |
| BI-RADS | 1.668 | 0.304 | 5.489 | 0.000 | 1.149 |  |
| original_shape2D_Elongation_modal1 | 6.176 | 2.104 | 2.935 | 0.003 | 2.849 |  |
| original_shape2D_Sphericity_modal1 | -8.213 | 2.702 | -3.040 | 0.002 | 2.742 |  |
| gradient_glrlm_RunVariance_modal1 | -6.573 | 2.749 | -2.391 | 0.017 | 1.569 |  |
| lbp-2D_firstorder_RobustMeanAbsoluteDeviation_modal1 | 4.370 | 2.201 | 1.986 | 0.047 | 1.213 |  |
| lbp-2D_glrlm_RunEntropy_modal1 | 10.221 | 2.230 | 4.583 | 0.000 | 1.398 |  |
| squareroot_glrlm_GrayLevelNonUniformityNormalized_modal1 | -4.411 | 2.079 | -2.122 | 0.034 | 1.125 |  |
| wavelet-HL_gldm_SmallDependenceEmphasis_modal1 | -6.456 | 3.864 | -1.671 | 0.095 | 1.274 |  |
| wavelet-HL_glszm_GrayLevelVariance_modal1 | -3.721 | 2.696 | -1.380 | 0.168 | 1.066 |  |
| wavelet-LL_firstorder_10Percentile_modal1 | 2.384 | 1.076 | 2.216 | 0.027 | 1.102 |  |
| *Intercept* is the cut-off value of the correction model and does not participate in VIF calculation; *Estimate* is the coefficient estimate of each independent variable, indicating the influence degree of the independent variable on the dependent variable | | | | | |  |

| **Table S7.** Performance index of univariate model of radiomics features based on *shape* semantic features | | | | | | | |
| --- | --- | --- | --- | --- | --- | --- | --- |
| **Feature** | **AUC**  **（95% CI）** | **ACC** | **SE** | **SP** | **BT** | **PPV** | **NPV** |
| original_shape2D_Elongation_modal1 | 0.510  （0.478-0.542） | 0.494 | 0.817 | 0.292 | 0.354 | 0.817 | 0.292 |
| original_shape2D_Sphericity_modal1 | 0.568  （0.536-0.601） | 0.662 | 0.437 | 0.802 | 0.424 | 0.437 | 0.802 |
| gradient_glrlm_RunVariance_modal1 | 0.471  （0.438-0.503） | 0.457 | 0.476 | 0.584 | 0.369 | 0.524 | 0.416 |
| gradient_ngtdm_Busyness_modal1 | 0.651  （0.622-0.681） | 0.646 | 0.706 | 0.609 | 0.409 | 0.706 | 0.609 |
| lbp-2D_firstorder_RobustMeanAbsoluteDeviation_modal1 | 0.518  （0.485-0.550） | 0.576 | 0.516 | 0.614 | 0.386 | 0.516 | 0.614 |
| lbp-2D_glrlm_RunEntropy_modal1 | 0.637  （0.608-0.667） | 0.598 | 0.857 | 0.436 | 0.303 | 0.857 | 0.436 |
| squareroot_glrlm_GrayLevelNonUniformityNormalized_modal1 | 0.513  （0.481-0.544） | 0.485 | 0.897 | 0.228 | 0.354 | 0.897 | 0.228 |
| wavelet-LH_gldm_DependenceVariance_modal1 | 0.472  （0.439-0.505） | 0.561 | 0.405 | 0.658 | 0.386 | 0.405 | 0.658 |
| wavelet-LH_glszm_LargeAreaHighGrayLevelEmphasis_modal1 | 0.531  （0.499-0.563） | 0.640 | 0.238 | 0.891 | 0.387 | 0.238 | 0.891 |
| wavelet-HL_gldm_SmallDependenceEmphasis_modal1 | 0.560  （0.529-0.591） | 0.555 | 0.706 | 0.460 | 0.374 | 0.706 | 0.460 |
| wavelet-HL_glszm_GrayLevelVariance_modal1 | 0.546  （0.514-0.579） | 0.613 | 0.381 | 0.757 | 0.382 | 0.381 | 0.757 |
| wavelet-LL_firstorder_10Percentile_modal1 | 0.521  （0.488-0.554） | 0.616 | 0.389 | 0.757 | 0.409 | 0.389 | 0.757 |
| log-sigma-3-0-mm-3D_firstorder_Maximum_modal1 | 0.514  （0.483-0.546） | 0.491 | 0.833 | 0.277 | 0.350 | 0.833 | 0.277 |
| *ACC* accuracy, *SEN* sensitivity, *SPE* specificity, *BT* best threshold, *PPV* positive predictive value, *NPV* negative predictive value, *95%CI* indicates 95% confidence interval | | | | | | | |

| **Table S8.** Performance index of univariate model of radiomics features based on *aspect ratio* semantic features | | | | | | | |  |
| --- | --- | --- | --- | --- | --- | --- | --- | --- |
| **Feature** | **AUC**  **（95% CI）** | **ACC** | **SE** | **SP** | **BT** | **PPV** | **NPV** | |
| original_shape2D_Elongation_modal1 | 0.752  (0.720-0.784) | 0.735 | 0.754 | 0.730 | 0.190 | 0.754 | 0.730 | |
| original_shape2D_Sphericity_modal1 | 0.564  (0.521- 0.704) | 0.704 | 0.443 | 0.764 | 0.208 | 0.443 | 0.764 | |
| gradient_glrlm_RunVariance_modal1 | 0.521  （0.479-0.563） | 0.509 | 0.738 | 0.457 | 0.182 | 0.738 | 0.457 | |
| gradient_ngtdm_Busyness_modal1 | 0.578  （0.539-0.616） | 0.677 | 0.525 | 0.712 | 0.190 | 0.525 | 0.712 | |
| lbp-2D_firstorder_RobustMeanAbsoluteDeviation_modal1 | 0.584  （0.544-0.624） | 0.680 | 0.525 | 0.715 | 0.196 | 0.525 | 0.715 | |
| lbp-2D_glrlm_RunEntropy_modal1 | 0.671  (0.634-0.707） | 0.683 | 0.721 | 0.674 | 0.193 | 0.721 | 0.674 | |
| squareroot_glrlm_GrayLevelNonUniformityNormalized_modal1 | 0.502  （0.456-0.548） | 0.780 | 0.295 | 0.891 | 0.189 | 0.295 | 0.891 | |
| wavelet-LH_gldm_DependenceVariance_modal1 | 0.600  （0.566-0.635） | 0.558 | 0.803 | 0.502 | 0.169 | 0.803 | 0.502 | |
| wavelet-LH_glszm_LargeAreaHighGrayLevelEmphasis_modal1 | 0.555  （0.518-0.592） | 0.552 | 0.770 | 0.502 | 0.185 | 0.770 | 0.502 | |
| wavelet-HL_gldm_SmallDependenceEmphasis_modal1 | 0.523  （0.483-0.564） | 0.543 | 0.590 | 0.532 | 0.181 | 0.590 | 0.532 | |
| wavelet-HL_glszm_GrayLevelVariance_modal1 | 0.609  （0.570-0.649） | 0.723 | 0.475 | 0.779 | 0.235 | 0.475 | 0.779 | |
| wavelet-LL_firstorder_10Percentile_modal1 | 0.473  （0.431-0.515） | 0.622 | 0.492 | 0.652 | 0.188 | 0.492 | 0.652 | |
| log-sigma-3-0-mm-3D_firstorder_Maximum_modal1 | 0.467  （0.426-0.507） | 0.595 | 0.459 | 0.625 | 0.188 | 0.459 | 0.625 | |
| *ACC* accuracy, *SEN* sensitivity, *SPE* specificity, *BT* best threshold, *PPV* positive predictive value, *NPV* negative predictive value, *95%CI* indicates 95% confidence interval | | | | | | | | |

| **Table S9.** Performance index of univariate model of radiomics features based on *boundary* semantic features | | | | | | | |  |
| --- | --- | --- | --- | --- | --- | --- | --- | --- |
| **Feature** | **AUC**  **（95% CI）** | **ACC** | **SE** | **SP** | **BT** | **PPV** | **NPV** | |
| original_shape2D_Elongation_modal1 | 0.656  （0.627-0.685） | 0.659 | 0.699 | 0.613 | 0.488 | 0.699 | 0.613 | |
| original_shape2D_Sphericity_modal1 | 0.474  （0.443-0.506） | 0.463 | 0.532 | 0.542 | 0.529 | 0.468 | 0.458 | |
| gradient_glrlm_RunVariance_modal1 | 0.447  （0.415-0.478） | 0.485 | 0.462 | 0.574 | 0.530 | 0.538 | 0.426 | |
| gradient_ngtdm_Busyness_modal1 | 0.631  （0.601-0.660） | 0.637 | 0.746 | 0.516 | 0.454 | 0.746 | 0.516 | |
| lbp-2D_firstorder_RobustMeanAbsoluteDeviation_modal1 | 0.487  （0.456-0.518） | 0.543 | 0.642 | 0.432 | 0.526 | 0.642 | 0.432 | |
| lbp-2D_glrlm_RunEntropy_modal1 | 0.692  （0.664-0.719） | 0.686 | 0.642 | 0.735 | 0.554 | 0.642 | 0.735 | |
| squareroot_glrlm_GrayLevelNonUniformityNormalized_modal1 | 0.581  （0.550 -0.611） | 0.604 | 0.798 | 0.387 | 0.496 | 0.798 | 0.387 | |
| wavelet-LH_gldm_DependenceVariance_modal1 | 0.486  （0.454-0.518） | 0.591 | 0.919 | 0.226 | 0.488 | 0.919 | 0.226 | |
| wavelet-LH_glszm_LargeAreaHighGrayLevelEmphasis_modal1 | 0.519  （0.487-0.550） | 0.555 | 0.416 | 0.710 | 0.518 | 0.416 | 0.710 | |
| wavelet-HL_gldm_SmallDependenceEmphasis_modal1 | 0.577  （0.545-0.608） | 0.634 | 0.838 | 0.406 | 0.499 | 0.838 | 0.406 | |
| wavelet-HL_glszm_GrayLevelVariance_modal1 | 0.580  （0.549-0.611） | 0.610 | 0.711 | 0.497 | 0.543 | 0.711 | 0.497 | |
| wavelet-LL_firstorder_10Percentile_modal1 | 0.616  （0.587-0.646） | 0.634 | 0.451 | 0.839 | 0.601 | 0.451 | 0.839 | |
| log-sigma-3-0-mm-3D_firstorder_Maximum_modal1 | 0.524  （0.493-0.555） | 0.558 | 0.618 | 0.490 | 0.508 | 0.618 | 0.490 | |
| *ACC* accuracy, *SEN* sensitivity, *SPE* specificity, *BT* best threshold, *PPV* positive predictive value, *NPV* negative predictive value, *95%CI* indicates 95% confidence interval | | | | | | | | |

| **Table S10.** Performance index of univariate model of radiomics features based on *edge* semantic features | | | | | | | | |
| --- | --- | --- | --- | --- | --- | --- | --- | --- |
| **Feature** | **AUC**  **（95% CI）** | **ACC** | **SE** | **SP** | **BT** | **PPV** | **NPV** | |
| original_shape2D_Elongation_modal1 | 0.585  （0.546-0.624） | 0.616 | 0.606 | 0.652 | 0.780 | 0.606 | 0.652 | |
| original_shape2D_Sphericity_modal1 | 0.475  （0.436-0.515） | 0.750 | 0.903 | 0.174 | 0.780 | 0.903 | 0.174 | |
| gradient_glrlm_RunVariance_modal1 | 0.495  （0.456-0.534） | 0.695 | 0.799 | 0.304 | 0.788 | 0.799 | 0.304 | |
| gradient_ngtdm_Busyness_modal1 | 0.596  （0.561-0.632） | 0.509 | 0.429 | 0.812 | 0.808 | 0.429 | 0.812 | |
| lbp-2D_firstorder_RobustMeanAbsoluteDeviation_modal1 | 0.488  （0.447-0.528） | 0.750 | 0.876 | 0.275 | 0.771 | 0.876 | 0.275 | |
| lbp-2D_glrlm_RunEntropy_modal1 | 0.650  （0.618-0.681） | 0.549 | 0.459 | 0.884 | 0.838 | 0.459 | 0.884 | |
| squareroot_glrlm_GrayLevelNonUniformityNormalized_modal1 | 0.449  （0.411-0.487） | 0.576 | 0.344 | 0.725 | 0.787 | 0.656 | 0.275 | |
| wavelet-LH_gldm_DependenceVariance_modal1 | 0.542  （0.501-0.583） | 0.756 | 0.876 | 0.304 | 0.722 | 0.876 | 0.304 | |
| wavelet-LH_glszm_LargeAreaHighGrayLevelEmphasis_modal1 | 0.542  （0.503-0.582） | 0.686 | 0.761 | 0.406 | 0.772 | 0.761 | 0.406 | |
| wavelet-HL_gldm_SmallDependenceEmphasis_modal1 | 0.545  （0.504-0.586） | 0.701 | 0.772 | 0.435 | 0.771 | 0.772 | 0.435 | |
| wavelet-HL_glszm_GrayLevelVariance_modal1 | 0.557  （0.518-0.595） | 0.674 | 0.734 | 0.449 | 0.792 | 0.734 | 0.449 | |
| wavelet-LL_firstorder_10Percentile_modal1 | 0.532  （0.496-0.567） | 0.451 | 0.351 | 0.826 | 0.815 | 0.351 | 0.826 | |
| log-sigma-3-0-mm-3D_firstorder_Maximum_modal1 | 0.473  （0.438-0.509） | 0.530 | 0.394 | 0.754 | 0.786 | 0.606 | 0.246 | |
| *ACC* accuracy, *SEN* sensitivity, *SPE* specificity, *BT* best threshold, *PPV* positive predictive value, *NPV* negative predictive value, *95%CI* indicates 95% confidence interval | | | | | | | |  |

| **Table S11.** Performance index of univariate model of radiomics features based on *calcification* semantic features | | | | | | | |  |
| --- | --- | --- | --- | --- | --- | --- | --- | --- |
| **Feature** | **AUC**  **（95% CI）** | **ACC** | **SE** | **SP** | **BT** | **PPV** | **NPV** | |
| original_shape2D_Elongation_modal1 | 0.454  （0.422-0.486） | 0.436 | 0.262 | 0.763 | 0.385 | 0.738 | 0.237 | |
| original_shape2D_Sphericity_modal1 | 0.571  （0.540-0.603） | 0.619 | 0.646 | 0.601 | 0.386 | 0.646 | 0.601 | |
| gradient_glrlm_RunVariance_modal1 | 0.509  （0.476-0.541） | 0.387 | 0.492 | 0.692 | 0.402 | 0.508 | 0.308 | |
| gradient_ngtdm_Busyness_modal1 | 0.544  （0.512-0.575） | 0.521 | 0.823 | 0.323 | 0.348 | 0.823 | 0.323 | |
| lbp-2D_firstorder_RobustMeanAbsoluteDeviation_modal1 | 0.515  （0.483-0.547） | 0.564 | 0.723 | 0.460 | 0.396 | 0.723 | 0.460 | |
| lbp-2D_glrlm_RunEntropy_modal1 | 0.560  （0.528-0.591） | 0.628 | 0.385 | 0.788 | 0.456 | 0.385 | 0.788 | |
| squareroot_glrlm_GrayLevelNonUniformityNormalized_modal1 | 0.498  （0.466-0.530） | 0.409 | 0.454 | 0.682 | 0.395 | 0.546 | 0.318 | |
| wavelet-LH_gldm_DependenceVariance_modal1 | 0.473  （0.440-0.505） | 0.579 | 0.346 | 0.732 | 0.396 | 0.346 | 0.732 | |
| wavelet-LH_glszm_LargeAreaHighGrayLevelEmphasis_modal1 | 0.500  （0.468-0.532） | 0.518 | 0.638 | 0.439 | 0.395 | 0.638 | 0.439 | |
| wavelet-HL_gldm_SmallDependenceEmphasis_modal1 | 0.526  （0.495-0.558） | 0.570 | 0.600 | 0.551 | 0.391 | 0.600 | 0.551 | |
| wavelet-HL_glszm_GrayLevelVariance_modal1 | 0.552  （0.520-0.584） | 0.598 | 0.554 | 0.626 | 0.412 | 0.554 | 0.626 | |
| wavelet-LL_firstorder_10Percentile_modal1 | 0.524  （0.492-0.555） | 0.500 | 0.877 | 0.253 | 0.345 | 0.877 | 0.253 | |
| log-sigma-3-0-mm-3D_firstorder_Maximum_modal1 | 0.501  （0.468-0.535） | 0.595 | 0.438 | 0.697 | 0.412 | 0.438 | 0.697 | |
| *ACC* accuracy, *SEN* sensitivity, *SPE* specificity, *BT* best threshold, *PPV* positive predictive value, *NPV* negative predictive value, *95%CI* indicates 95% confidence interval | | | | | | | | |
